# Exploration of Zr–Metal–Organic Framework as Efficient Photocatalyst for Hydrogen Production

**DOI:** 10.1186/s11671-017-2311-6

**Published:** 2017-09-19

**Authors:** Zhiliang Jin, Hao Yang

**Affiliations:** School of Chemistry and Chemical Engineering, North Minzu University, Yinchuan, 750021 People’s Republic of China

**Keywords:** Zr–metal–organic framework, UiO-66, Hydrogen production, Photocatalyst

## Abstract

The application of metal–organic framework (MOF) in the field of photocatalysis is limited, because of its unstable chemical properties and failure to respond in visible light. Herein, the Pd/MOF catalysts were prepared by impregnation reduction. It is important that we have reasonably constructed the dye-sensitized system of Pd/MOF and successfully extended the application of MOF to the visible range. It exhibited maximal photocatalytic activity (9.43 mmol/g) under visible-light irradiation (*λ* ≥ 420 nm) with eosin Y as a photo-sensitizer, which was enhanced twice the order of magnitude compared with the pure MOF (0.03 mmol/g). The activation process with an exchangeable guest solvent produced the Zr–MOF with the high surface area and the high stability, which provide a great electronic transmission capacity. The Pd nanoparticles provide an electronic outlet, and the dye broadens the spectral absorption range. The synergistic effect of the various components contributes to the high hydrogen production activity. This work provides a reference for the application of MOF in the field of photocatalysis.

## Background

Energy crisis and environment pollution problem received more and more attention due to inefficient use of vast fossil fuels [1]. Therefore, the search for renewable energy alternatives to fossil fuels is a highly significant yet challenging assignment [2–4]. Photocatalytic hydrogen production through water splitting by using solar energy has been considered as an alternative environmentally benign way, but it is limited to develop highly active and stable photocatalysts at low cost. So far, various photocatalysts have been developed for H_2_ production, for instance, metal oxide (TiO_2_ [5–7], NiO [8], CuO [9]), metal sulfide (CdS [10], CuS [11], NiS [12], MoS [13, 14]), and nitride semiconductors (C_3_N_4_) [15–22]. Recently, Yu and coworkers have made a systematic analysis of the heterojunction photocatalysts, which provides an important reference for the subsequent photocatalytic research [23]. Furthermore, some phosphorus such as CoP [24, 25], Ni_2_P [26], Cu_3_P [27], and MoP [28] have been employed for H_2_ production. Especially, metal–organic frameworks (MOFs) with outstanding characteristics have attracted tremendous attention as catalysts or catalyst carriers for photocatalytic water splitting in the past few years [29–31]. Although there are still some limitations, the opportunities for MOFs as heterogeneous catalysts are very encouraging [32–35] because MOFs as a new class of porous material have exciting characteristics, such as high surface areas, crystalline open structures, tunable pore size, and functionality. Particularly, the high specific surface area of MOFs may provide more attachment points for a cocatalyst, in which it could create more active sites and make better contact with reactants. On the other hand, the porous structures of MOFs can also provide extra pathways for the migration of photo-induced electrons and facilitate separation of the charge carrier [36, 37]. For all these reasons, MOFs as a highly efficient photocatalyst could be expected. More importantly, some photoactive MOFs have been reported; specifically, NH_2_-MIL-125(Ti) could photocatalytically reduce CO_2_ to form formate under visible light according to Sun et al. [38]. Metal–organic framework and metal molecular are applied to spatial charge separation for enhancing H_2_ evolution under visible light [39].

Horiuchi et al. synthesized the amino-functionalized Ti-MOF-NH_2_, which exhibited efficient photocatalysis for hydrogen production reaction with triethanolamine (TEOA) as a sacrificial electron donor under visible-light irradiation condition [40]. Jiang et al. structured electron trap states in a metal–organic framework to enhance separation of electron–hole, which applied visible-light photoreduction of CO_2_ [41].

Moreover, the palladium nanoparticles were successfully encapsulated in UiO-66 material by Dong et al., which exhibited efficient catalytic activity for the Suzuki–Miyaura coupling reactions at mild condition [42].

Lillerud et al. first synthesized Zr-based MOFs, which they designated as UiO-66 [43]. It was reported that UiO-66 displayed high chemical and thermal stability (500 °C) owing to its high affinity of zirconium towards oxygen ligands and the compact structure [44]. In 2010, Garcia and coworkers used UiO-66 for water splitting under UV light irradiation, which opened the door for MOFs to photocatalytic water splitting [45]. However, the UiO-66 cannot be effective for photocatalysis because they cannot respond to visible light effectively. It is well known that dye-sensitized photocatalysts could expand absorption band edge and intensity, typically a metal complex photosensitizer. In 2014, He and coworkers described the application of UiO-66 and Pt@UiO-66, using rhodamine B as photosensitizer, in these systems, and the catalytic activities were 33 and 116 μmol g^−1^ h^−1^, respectively [46]. This work successfully extended the range of UiO-66 absorption to visible light, but the activity of hydrogen production of the system was still low. In 2015, Yuan and coworkers described a very simple system using erythrosine B dye to sensitize UiO-66 and the highest H_2_ production rate of 4.6 μmol h^−1^ [47]. In 2016, Xiao and coworkers described a Pt@UI-66-NH_2_ catalytic system with a very high hydrogen production activity (257.38 μmol g^−1^ h^−1^) [48]. The synergistic of metal–organic frameworks and metal nanoparticles for enhanced catalysis is systematically reviewed in Jiang’ work [49, 50].

UiO-66 which has an absorption band edge of 335 nm in the UV region could be attributed to the π − π* transition in organic ligands. UiO-66 exhibits photocatalytic activity because of its ability to act like a semiconductor [45]. Although UiO-66 has been widely studied in the field of photocatalysis, its photocatalytic efficiency is still very low. Therefore, there is still a long way to go to find the proper cocatalyst and construct a reasonable photocatalytic system to enhance the photocatalytic activity of UiO-66 and application of photocatalysis.

In this work, we designed and constructed a dye-sensitized photocatalysis reaction system, where we use TEOA as a sacrificial donor under visible-light irradiation (*λ* ≥ 420 nm) and introduced eosin Y (EY) as a photosensitizer. A nanosize Zr–metal–organic framework (Zr-MOF, UiO-66) was solvothermally synthesized, and the Pd/MOF catalysts were prepared by impregnation reduction as well. The Pd-loaded Zr-MOF was tested for efficient photocatalytic hydrogen production. It exhibited maximal photocatalytic activity (2.28 mmol h^−1^ g^−1^) under visible-light irradiation (*λ* ≥ 420 nm) with EY as a photosensitizer.

## Methods

### Preparation of UiO-66

All chemicals were analytical grade and used directly without any further purification.

UiO-66 was synthesized via a solvothermal route. In a typical synthesis, ZrCl_4_ (1.89 g,) and terephthalic acid (2.79 g) were dissolved in 48.7 mL *N*,*N*-dimethylformamide (DMF) containing 1.43 mL hydrochloric acid (HCl) and then the solution was transferred to a 100 mL Teflon-lined stainless steel autoclave. The autoclave was sealed and heated in an oven at 220 °C for 20 h. After cooling naturally, the product was collected by centrifugation and washed for three times with DMF and then sequentially purified within methanol for several times to make sure that the occluded DMF molecules were eliminated. Finally, it followed by drying under vacuum (90 °C, 6 h) before using the samples for the photocatalytic reactions.

### Synthetic of Pd/MOF Composite Photocatalysts

The Pd/MOF composite photocatalysts were prepared by impregnation reduction [42, 50]. The above-prepared UiO-66 powders (0.2 g) were placed into a clean beaker, which contained 200 mL of deionized water and mixed with appropriate amount of H_2_PdCl_4_ solution, and were vigorously stirred for another 1 h to disperse them completely. Then the NaBH_4_ (freshly prepared) was added drop-wise into the solution with a continuous magnetic stirring; the reaction solution was kept on stirring for 3 h to complete the reduction reaction (*n*_(H2PdCl4)_:*n*_(NaBH4)_ = 1:3). The obtained black granules were washed with deionized water and dried in a vacuum oven at 70 °C for 6 h. H_2_PdCl_4_ was added in an amount of 1, 3, and 5% of the mass of UiO-66, and the resulting samples were named Pd/MOF 1%, Pd/MOF 3%, and Pd/MOF 5%, respectively.

### Characterization

Morphology of the sample was characterized by a field emission scanning electron microscope (JSM-6701F.JEOL) at an accelerating voltage of 5 kV. Transmission electron microscopy (TEM) measurements were taken on by using a FEI Tecnai TF20 microscope at 200 kV. The crystalline structure of the products was identified by X-ray diffraction analysis (XRD, Rigaku RINT-2000) using Cu Kα radiation at 40 keV and 40 mA. The XRD patterns were recorded from 10° to 90° with a scanning rate of 0.067°s^−1^. UV-vis diffuse reflectance spectra were taken on an UV-2550 (Shimadzu) spectrometer by using BaSO_4_ as the reference. The element composition was detected by X-ray photoelectron spectroscope (XPS, ESCALAB 250Xi). The nitrogen adsorption–desorption isotherms of samples were measured at 77 K with an ASAP 2020M instrument and analyzed by the Brunauer–Emmett–Teller (BET) equation. The pore size distribution plots were obtained by the Barret–Joyner–Halenda (BJH) model.

### Photocatalytic H_2_ Evolution Experiments

Photocatalytic experiments were conducted in a quartz glass reactor ca. 62 cm^3^, and the opening of the reactor was sealed with a silicone rubber septum. In a typical photocatalytic experiment, 10 mg of catalyst was suspended in 30 mL 15% (*v*/*v*) TEOA aqueous solution containing 20 mg dye EY and dispersed by means of ultrasonication for about 15 min. The system was degassed by bubbling N_2_ gas to ensure the reactant mixtures under anaerobic conditions. The system was irradiated by a 5-W light-emitting diode lamp (420 nm) for H_2_ evolution under magnetic stirring condition.

The amount of hydrogen evolution was measured using gas chromatography (Tianmei GC7900, TCD, 13 X columns, N_2_ as carrier).

### Photoelectrochemical Measurements

#### Preparation of Working Electrode

The fluorine-doped tin oxide (FTO) (1 × 5 cm^2^) substrate is washed by cleaning agent, acetone solution, isopropyl alcohol, ethanol, and water under ultrasonic processing for about 30 min. The catalyst (10 mg) was added in 500 μL anhydrous ethanol (containing 50 μL 5% Nafion solution) and ultrasonicated treatment to form suspension liquid. Subsequently, 0.2 mL of the above suspension is uniformly applied to the pre-treated FTO with a drip coating, and the coating area is controlled at about 1 cm^2^. The painted electrode is dried in natural environment, and the working electrode is obtained.

All PEC measurements were finished on a electrochemical workstation (VersaStat4-400, Advanced Measurement Technology, Inc.) in a three-electrode system using the as-prepared photoanode as the working electrode, a Pt plate as the counter electrode, and a saturated calomel electrode (SCE) as the reference electrode. A 300-W xenon lamp equipped with a filter (*λ* ≥ 420) was used as the irradiation source. 0.2 M Na_2_SO_4_ aqueous and hydrogen producing system solution (containing TEOA and dye EY) was employer as the electrolyte. Photocurrent response test of the photoanodes with on and off cycles were carried out at a fixed bias of 0.4 V vs. SCE.

## Results and Discussion

### Morphology and Structure

**Fig. 1:**
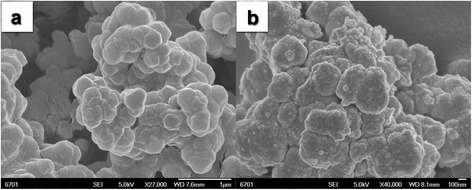
The typical scanning electron microscopy (SEM) patterns of **a** UiO-66 and **b** Pd/MOF

The typical scanning electron microscopy (SEM) imaging of the UiO-66 and Pd/MOF has been shown in Fig. 1. The original UiO-66 was prepared by a simple and mild solvothermal method. The SEM images (Fig. 1a) clearly displayed that the bare UiO-66 presents uniform size and smooth surface. Pd nanoparticles were obtained by reduction of H_2_PdCl_4_ at room temperature with sodium borohydride (NaBH_4_). Figure 1b is the SEM image of the Pd/MOF composite photocatalysts, which showed that the original smooth UiO-66 surface appeared with a lot of particulate matter. It can be proven that these small particles are elementary palladium by the subsequent XPS results. The glossy surface can provide extra pathways for photogenerated charges, and Pd nanoparticles on surface of UiO-66 can provide active sites for H_2_ evolution. The photogenerated charges can rapidly transfer from excited dye to UiO-66, and the enriched electrons on the UiO-66 can be transferred to the Pd nanoparticles and combined with the protons in the solution to form hydrogen molecules. It is beneficial for the separation of photogenerated electron–holes and for the enhancement of photocatalytic hydrogen production activity. Therefore, the high-efficiency hydrogen production by water splitting using visible light was expected.

**Fig. 2:**
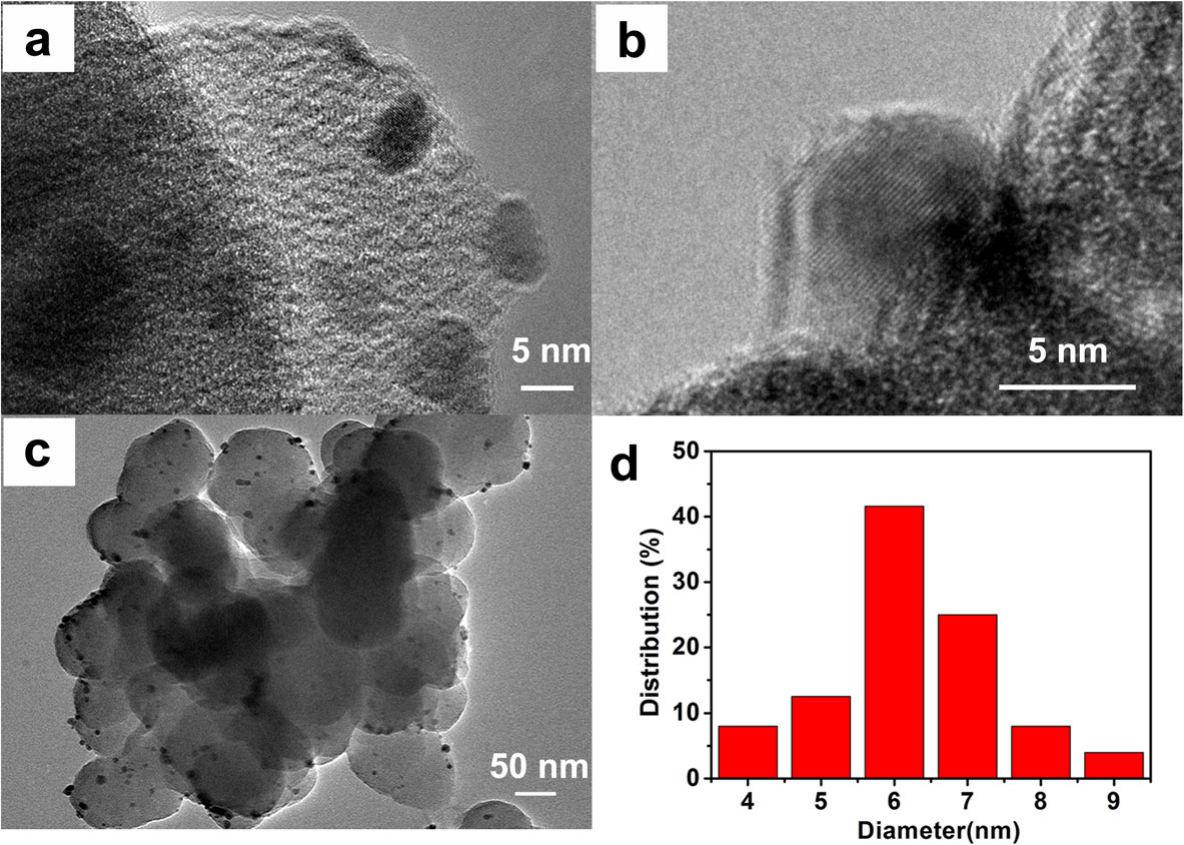
**a** The TEM patterns and **b** the HRTEM patterns of Pd/MOF 3% sample. **c**, **d** The size distribution for Pd NPs of Pd/MOF 3% sample

For further study of the morphology of Pd/MOF sample and the Pd nanoparticles of UiO-66 surface, the typical TEM image and the high-resolution TEM (HRTEM) image patterns were exhibited in Fig. 2. As shown in Fig. 2a, some of the Pd nanoparticles are exposed to the outside of the UiO-66, which is conducive to the binding of electrons and protons in the solution. In addition, it can be seen that the Pd nanoparticles are well dispersed on the surface of UiO-66 and the diameter is about 6 nm. The HRTEM image (Fig. 1b) clearly shows that the lattice spacing of the quantum-sized Pd nanoparticles is ca. 0.223 nm, which is consistent with the lattice spacing of the (111) plane of metallic Pd [48]. It can be clearly seen that the Pd nanoparticles were uniformly distributed on the surface of UiO-66 (Fig. 2c) and the size of the Pd nanoparticles is between 4 and 9 nm (Fig. 2d). In particular, the size of the Pd nanoparticles is mainly concentrated at 6 nm.

**Fig. 3:**
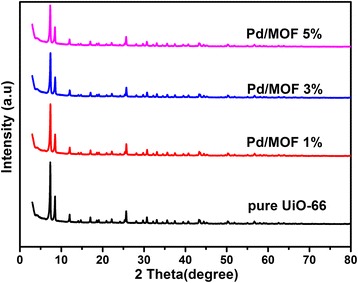
The X-ray diffraction (XRD) patterns of pure UiO-66, Pd/MOF 1%, Pd/MOF 3%, and Pd/MOF 5%

The X-ray diffraction (XRD) patterns of the pure UiO-66 and Pd/MOF are exhibited in Fig. 3. It is clearly shown that diffraction peaks are well indexed to other works, which indicated the MOF was successfully synthesized [43]. Moreover, it have been reported that the UiO-66 framework is stable in water, benzene, ethanol, and DMF, as well as in a strong acid (HCl) solution and a strong base (NaOH) solution where it still maintains an appreciable degree of crystallinity [43]. The patterns in Fig. 3 showed that the crystallinity of UiO-66 did not change after adding the Pd nanoparticles, which manifested the Pd nanoparticles have not destroyed the chemical structure of the framework. Therefore, the UiO-66 as a photocatalyst carrier is feasible and the stable production of hydrogen can be expected by water splitting using solar energy in the TEOA aqueous solution. In addition, the diffraction peaks of Pd were not observed in patterns because of low load, quantum size, and good dispersion.

**Fig. 4:**
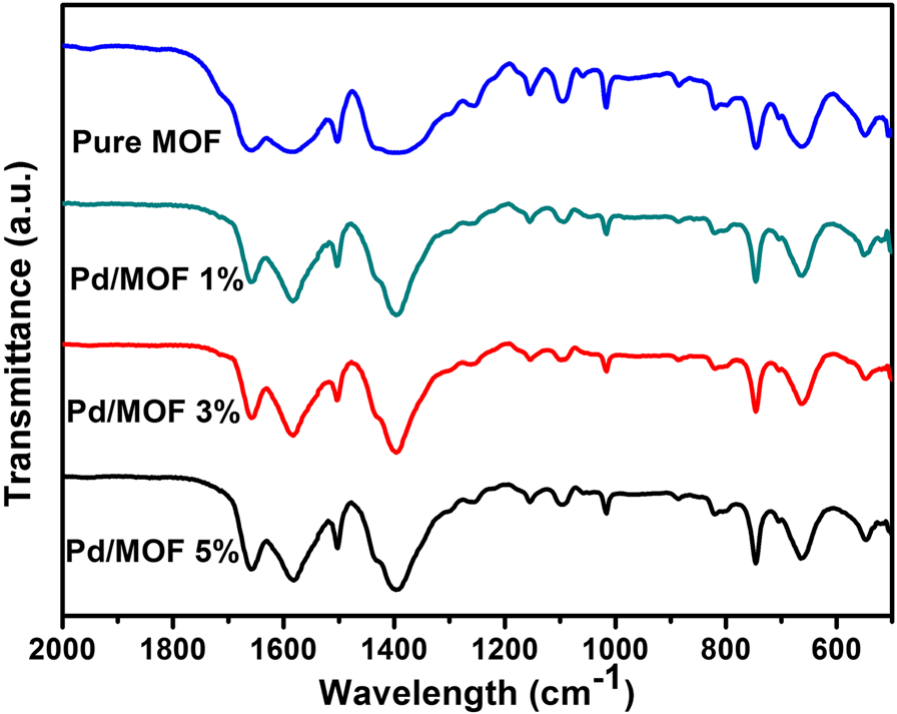
The Fourier transform infrared spectra (FTIR) of pure UiO-66, Pd/MOF 1%, Pd/MOF 3%, and Pd/MOF 5%

The Fourier transform infrared spectra (FTIR) of samples are showed in Fig. 4. The appeared shark peaks at 1585 and 1400 cm^−1^ are ascribed to the in- and out-of-phase stretching modes of the carboxylate group. Concretely, the peak at 1585 cm^−1^ is associated with C–C in the aromatic compound of the organic linker, and the peak at 1400 cm^−1^ is due to C–O root in C–OH of carboxylic acid. The peak at 659 and 746 cm^−1^ are associated with the O–H bending and Zr–O modes, respectively. Moreover, the peak at 552 cm^−1^ which is associated with Zr–(OC) symmetric stretching and the peak at 546 cm^−1^ which is associated with Zr–(OC) asymmetric stretching appeared at lower frequencies. In addition, the positions of each peak in all samples were not changed, indicating that the introduction of Pd nanoparticles did not destroy the chemical structure of UiO-66 [51].

**Fig. 5:**
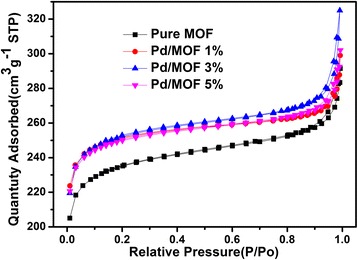
The N_2_ adsorption–desorption isotherms of pure MOF, Pd/MOF 1%, Pd/MOF 3%, and Pd/MOF 5%

The BET specific surface area (*S*_BET_) and the pore size distributions for samples were calculated by nitrogen adsorption–desorption isotherm measurements (Fig. 5). The pore size distributions were obtained by using desorption data by the BJH method. As shown in Table 1, it shows that the *S*_BET_ of pure UiO-66 is 791.6141 m^2^/g. After introducing Pd nanoparticles, the *S*_BET_ of the composite catalysts are increase in varying degrees. The sample of Pd/MOF 3% shows the largest *S*_BET_, which contributes to adsorption of dye molecule. In particular, the *S*_BET_ is decreased when the Pd nanoparticles continue to increase (Pd/MOF 5%, 838.9649 m^2^/g).

**Table 1:** The *S*_BET_, pore volume, and pore diameter of the four samples

Samples	*S*_BET_ (m^2^/g)	Pore volume (cm^3^/g)	Pore diameter (nm)
Pure MOF	791.6141	0.4162	2.1035
Pd/MOF 1%	843.0173	0.4451	2.1121
Pd/MOF 3%	847.4350	0.4775	2.2539
Pd/MOF 5%	838.9649	0.4524	2.1568

### X-ray Photoelectron Spectroscopy Patterns of Pd/MOF 3%

**Fig. 6:**
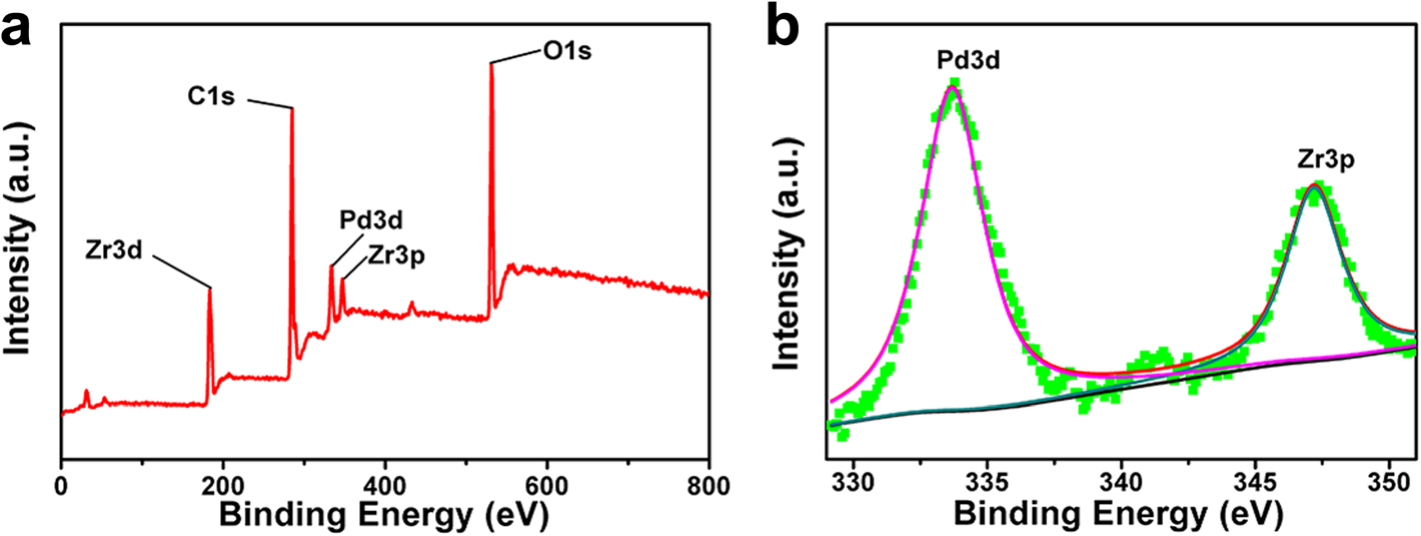
**a**, **b** X-ray photoelectron spectroscopy (XPS) patterns of Pd/MOF 3%

In order to determine the chemical composition and identify the chemical state of the elements in the sample, XPS spectra are also presented in Fig. 6. Specifically, all the peaks corresponding to the Zr, Pd, O, and C elements can be detected in Fig. 6a. Moreover, the high-resolution spectrum of the sample shows two peaks at 333.7 and 347.2 eV, which could be ascribed to Pd 3d and Zr 3p of metallic Pd. Those results demonstrated metallic Pd has been successfully deposited on the UiO-66 framework by reducing agent of NaHP_4_, which was in accordance with the previous results.

### The UV-Vis Absorption Spectra

**Fig. 7:**
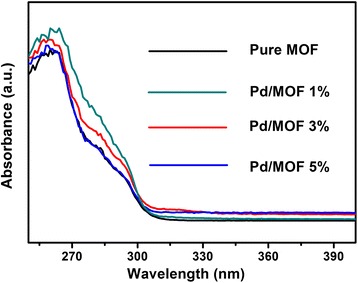
The UV-vis absorption spectra of pure UiO-66, Pd/MOF 1%, Pd/MOF 3%, and Pd/MOF 5%

It was already reported that the conduction band of UiO-66 was −0.6 V vs. normal hydrogen electrode (NHE), and this is more negative than the redox potential of H^+^/H_2_(−0.4 V vs. NHE, pH = 7). It proved that the original UiO-66 has the potential to produce hydrogen by water splitting [42]. However, Fig. 7 showed that the pure UiO-66 is a white powder with absorption band edge of only 300 nm and the Pd/MOF band edge of optical absorption has any evident changes after adding metallic Pd, as a result, all of the bare MOF and Pd/MOf could not produce hydrogen by water splitting in the visible-light region. In order to resolve this issue, EY was employed for increasing the region of absorption. It is known that EY is a photosensitizer, which could absorb visible light. Therefore, photocatalytic water splitting for hydrogen production using visible light is entirely feasible.

### The Photocatalytic Activities of Different Catalysts for Hydrogen Evolution

The process of photocatalytic activities for hydrogen evolution have been designed and implemented, which used TEOA (pH 10) as a sacrificial donor under visible-light irradiation (*λ* ≥ 420 nm) and introduced EY as a photosensitizer. The time courses of H_2_ evolution over different catalysts indicated that only trace amount of H_2_ was detected (only 0.03 mmol after 4-h irradiation) in the pure UiO-66, which is due to the electron of excited EY which could not outright react with H^+^ in the absence of metallic palladium. With the addition of different amounts of Pd nanoparticles, the photocatalytic hydrogen production increased in different degrees. 3.24 mmol of H_2_ was produced after 4 h of irradiation with 1% Pd loaded to MOF. It suggested that Pd was an active species for H_2_ production. Furthermore, when 3% Pd was loaded to MOF, 9.43 mmol of H_2_ evolution evolved, which attributes to more active sites obtained. However, 6.06 mmol of H_2_ evolution was produced after loading 5% Pd, in which because superfluous Pd nanoparticles covered the surface of UiO-66, the absorption of dye molecules on UiO-66 was hindered. We tested the adsorption of different dyes on the catalyst. The experimental results were consistent with the BET testing data. The sample of Pd/MOF 3% showed the maximum dye adsorption (39.7 μmol/g) (Table 2). The affinity of metal Pd and UiO-66 for dye molecules is different, so excessive Pd loading to surface of UiO-66 prevents adsorption of the dye molecule. On the other hand, the sample Pd/MOF 5% exhibited a reduced *S*_BET_, which may also lead to a decrease in the amount of dye adsorbed.

**Table 2:** The adsorption of eosin Y on various samples

Samples	Uio-66	Pd/MOF 1%	Pd/MOF 3%	Pd/MOF 5%
Amount of adsorbed eosin (μmol/g)	30.1	34.3	39.7	34.6

### Effect of the pH on the Photocatalytic Activity Pd/MOF 3% of (10 mg) for Hydrogen Evolution (Reaction Time 5 h)

**Fig. 8:**
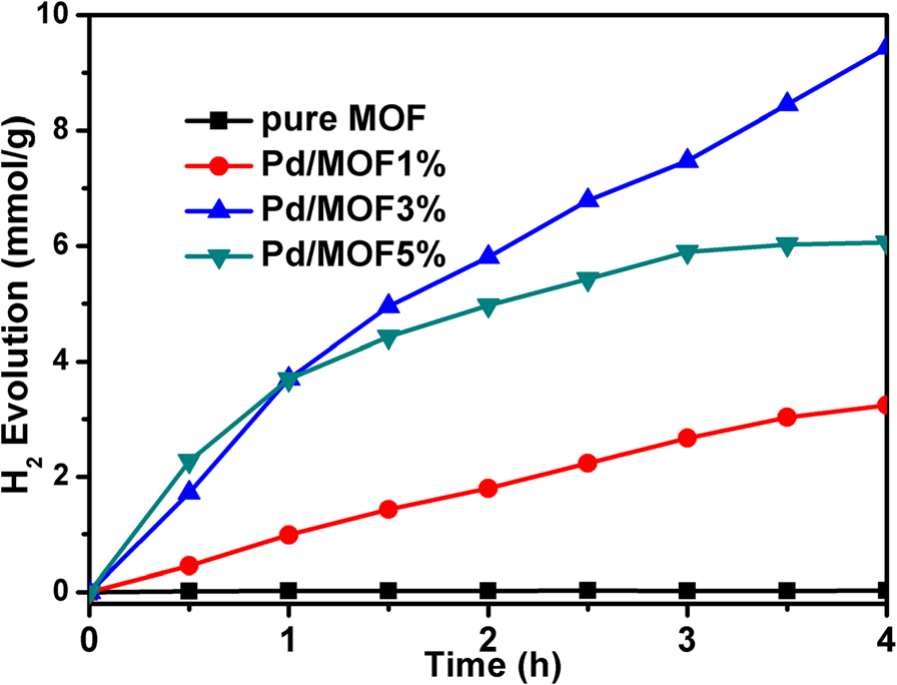
The photocatalytic activities of pure MOF, Pd/MOF 1%, Pd/MOF 3%, and Pd/MOF 5% for hydrogen evolution

**Fig. 9:**
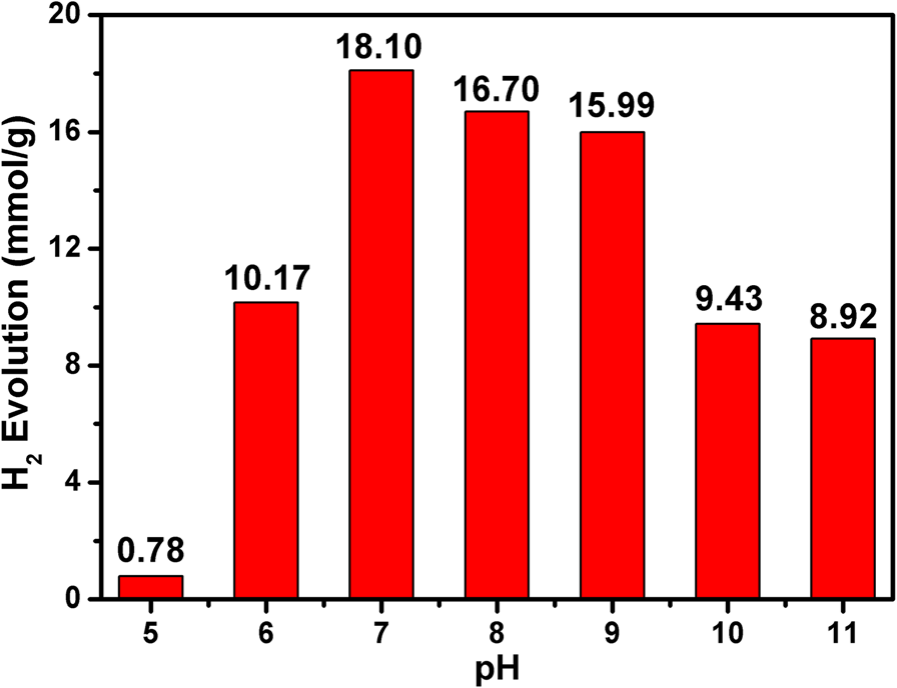
Effect of the pH on the photocatalytic activity of Pd/MOF 3% (10 mg) for hydrogen evolution (reaction time 5 h, EY 20 mg)

The solution pH had significant influence on photocatalytic activity (Figs. 8 and 9) [52]. Different pH values of the solution were studied from 5 to 11. It can be distinctly seen that the rate of hydrogen evolution maximized at pH 7 (18.10 mmol), while the rate of H_2_ evolution was decreased under both more acidic and more alkaline TEOA aqueous solutions. The rate of hydrogen evolution was only 0.78 mmol at pH 5 because of the protonation of TEOA at acidic pH, which resulted in a shorter lifetime and efficiency of excited EY, and the rate of hydrogen evolution was decreased. However, the activity of the photocatalyst exhibited a decrease following increase of the basicity. This is because the strong alkaline conditions reduce the concentration of H^+^ and lead to a decrease in the kinetics of hydrogen production.

### The Stability Testing for the Sample of Pd/MOF 3% in the Dye-Sensitized Photocatalytic System

**Fig. 10:**
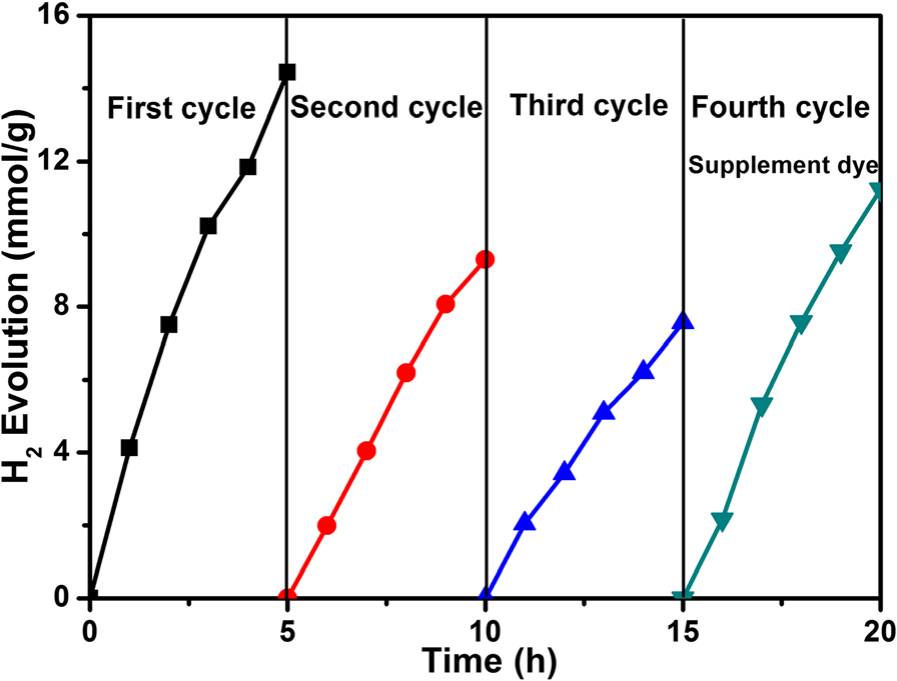
The stability testing for the sample of Pd/MOF 3% in the dye-sensitized photocatalytic system

In order to verify the stability of the system, a stability test was carried out. The stability experiment was carried out in TEOA aqueous solution adding EY as sensitizer. As shown in Fig. 10, the experiment was implemented in four stages. In the first cycle, the hydrogen production rate increased persistently in 5 h, which is because the dye has played a key role in the first cycle of reaction. In the second cycle, the N_2_ is used to replace the gas in the reaction system without any additional dyes. At this stage, hydrogen production is reduced, which is due to the dye that was degraded with the reaction time increasing. In the third stage, the N_2_ replacement gas is used as the second stage and no dye is added. At this stage, the hydrogen production decreased continuously, and this was mainly due to the addition of degraded dyes. In the fourth stage, the gas in the reaction system was substituted with N_2_ and added to EY (20 mg). At this stage, the yield of hydrogen is markedly recovered compared to the third phase, which is due to the eosin that was added. The above results show that the prepared Pd/MOF 3% catalyst has excellent properties, and the stability of the dye-sensitized system needs to be improved, which is also the focus of the next step.

### Photoluminescence (PL) Analysis

**Fig. 11:**
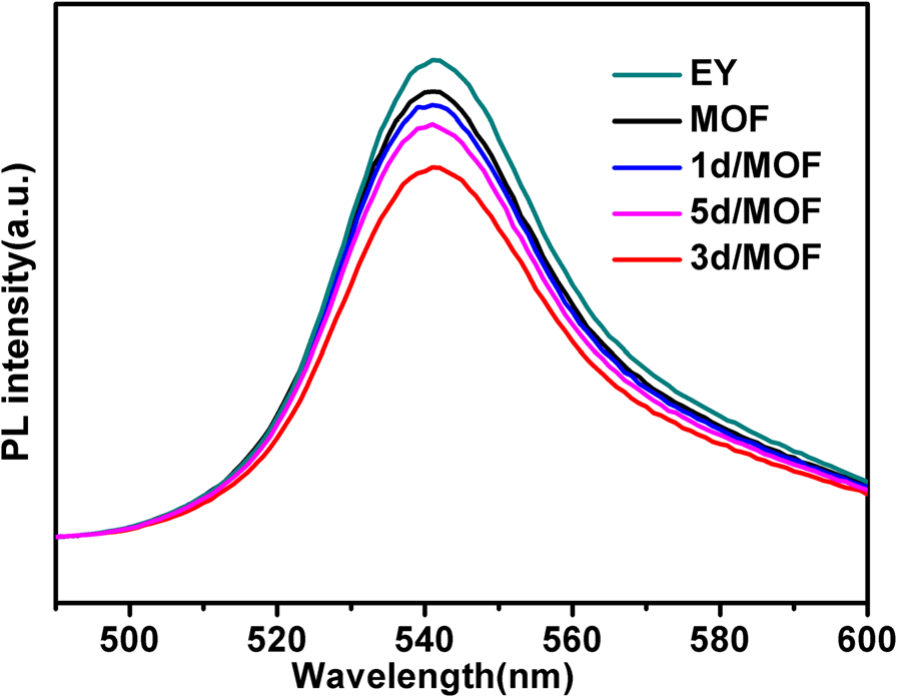
Fluorescence spectra of the EY-sensitized MOF, Pd/MOF 1%, Pd/MOF 3%, and Pd/MOF 5% samples in 15% (*v*/*v*) TEOA aqueous solution at pH 10

In order to investigate the transfer of photo-generated electrons, the photoluminescence quenching of EY were examined. As shown in Fig. 11, the dye EY showed up a maximum emission wavelength of 537 nm when the excitation wavelength was 460 nm. After the addition of UiO-66, the intensity of the maximum excitation wavelength did not obviously change, but the fluorescence intensity of the maximum emission wavelength was reduced in different degrees after adding different Pd/MOF samples. This indicates that Pd particles play a key role in the electron transfer of the system. When the Pd/MOF 3% was added, the fluorescence intensity was the lowest, which proved the best separation effect of photogenerated electron hole. This result was in accordance with the time courses of hydrogen evolution by different catalysts. When Pd nanoparticles have been introduced, the photo-generated electrons in UiO-66 could rapidly transfer to Pd. Thus, they exhibited a sharp decrease in emission intensity of EY.

### The Photoelectric Performance Testing

**Fig. 12:**
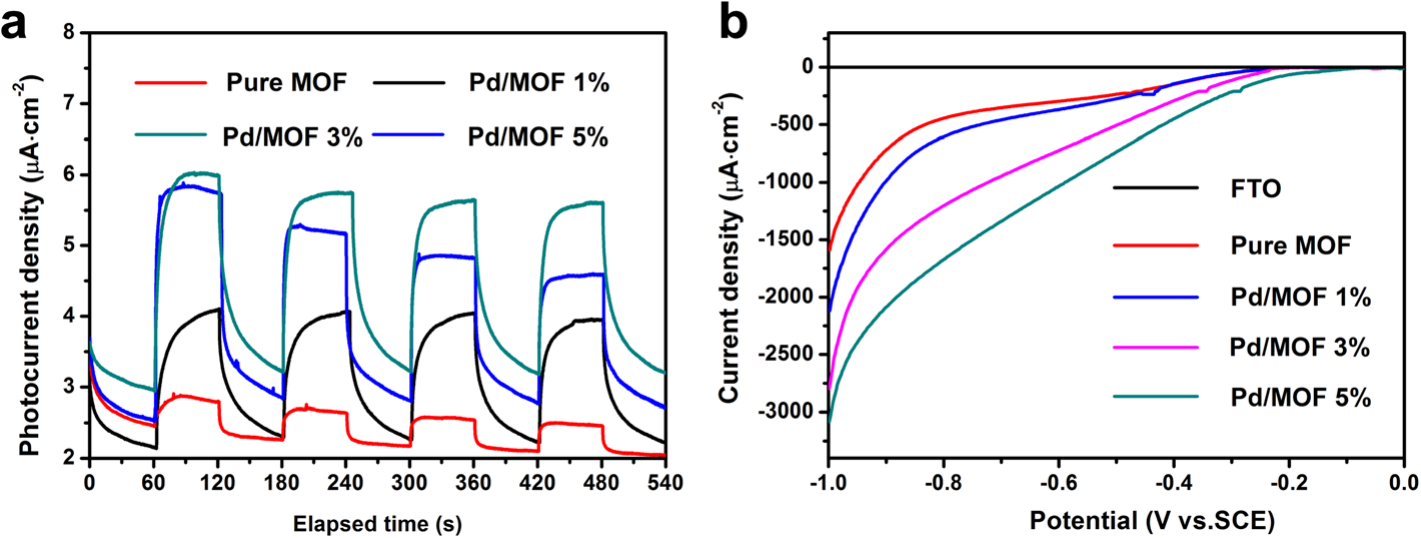
**a** Transient photocurrent responses for samples of MOF, Pd/MOF 1%, Pd/MOF 3%, and Pd/MOF 5% in 0.2 M Na_2_SO_4_ aqueous solution under visible-light irradiation (*λ* ≥ 420 nm) at 0.4 V vs. SCE. **b** Linear sweep voltammograms of MOF, Pd/MOF 1%, Pd/MOF 3%, and Pd/MOF 5% in 0.2 M Na_2_SO_4_ aqueous solution under visible-light irradiation (*λ* ≥ 420 nm)

In order to further study the electron transfer process in the system, the photoelectric property of the dye-sensitized photocatalytic hydrogen production system was also tested. Figure 12a is the instantaneous photocurrent–time curves for samples of pure MOF, Pd/MOF 1%, Pd/MOF 3%, and Pd/MOF 5%. It shows that the photocurrent density of pure MOF is lowest under visible light. When the Pd nanoparticles were loaded onto MOF, the photocurrent density increased markedly, and when the 3% Pd was introduced, the photocurrent density reached the maximum. The superior specific surface area of MOF material is favorable for the adsorption of a large number of dye molecules. Under visible-light irradiation, dye EY is excited to form an excited state and then the excited state EY molecule is quenched by TEOA to form a strong reducing EY^−^·. EY^−^· can rapidly deliver electrons to the MOF and then participate in the reactions. However, electrons cannot be rapidly involved in the transfer process, resulting in the loss of a large number of electrons. Thus, a single MOF shows very low photocurrent densities and hydrogen production activity. Pd nanoparticles serve as an electron outlet, enabling the rapid transfer of large amounts of electrons to the MOF surface and thereby enhancing the photocurrent density and hydrogen production. The photoelectric test results are in good agreement with the hydrogen production kinetics, and it proved that the Pd nanoparticles can indeed enhance the photocatalytic activity of MOF.

Figure 12b is a linear voltammetric scan for the samples. As can be seen from the diagram, a weak current response is found on the FTO at the lower voltage, which is due to the cathodic current produced by the reduction of H^+^ to H_2_ under severe negative pressure. At the same voltage, the sample adding Pd showed a higher current response than the pure MOF. Especially after 3% of the Pd was added to MOF, the sample Pd/MOF 3% showed the most prominent current response. These results are in good agreement with the above test results. It proved that Pd nanoparticles can significantly enhance the hydrogen production activity of MOF in dye-sensitized photocatalytic system.

### Speculation on the Mechanism for H_2_ Evolution

**Scheme 1:**
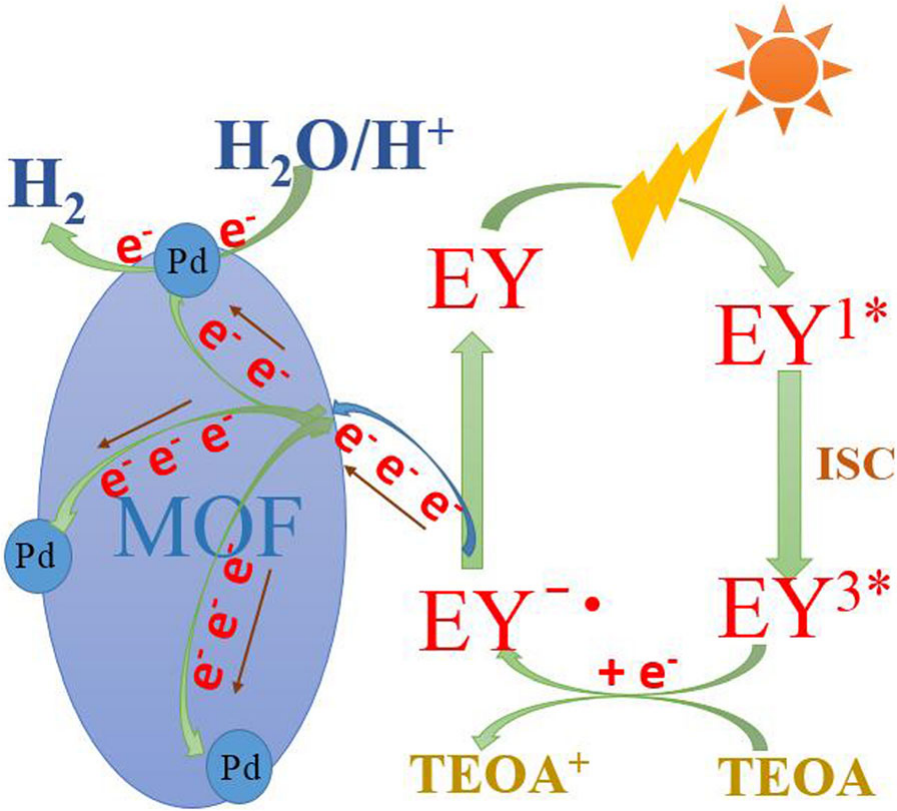
Possible mechanism of photocatalytic hydrogen evolution in EY-sensitized system

Based on the above results, the possible mechanism of photocatalytic hydrogen evolution in EY-sensitized system can be explained in Scheme 1. The large specific surface area of UiO-66 is beneficial to the adsorption of dye molecules. Firstly, a large number of EY molecules were adsorbed onto the surface of UiO-66 by physical adsorption. Then, the ground state of EY absorbs light photo to form singlet excited state EY^1*^under visible-light irradiation. Singlet excited EY^1*^ rapidly produces the lowest lying triplet excited state EY^3*^ via an efficient intersystem crossing (ISC). In the case of existing TEOA as a sacrificial donor, EY ^3*^can be reductively quenched and can produce EY^−·^. Subsequent electron transfer can be obtained by comparing the energy levels. It has been reported that the reductive potential of EY^−·^ is −0.8 V vs. NHE, and the conduction band of UiO-66 is −0.6 V vs. NHE. Therefore, the electrons can actively transmit from EY^−·^ to UiO-66. The accumulated electrons on UiO-66 frameworks will transfer to the Pd nanoparticles, and finally, H^+^ obtains electrons from Pd to form hydrogen. UiO-66 can function as an excellent electron acceptor and transporter to efficiently prolong the lifetime of charge carriers. Consequently, it improves the charge separation efficiency and the catalytic H_2_ evolution activity of the Pd/MOF. The UiO-66 has extremely large surface area and well-order porous structures and channels and is conducive to the electron transfer.

## Conclusions

The Pd/MOF catalysts were prepared by impregnation reduction. It is important that we have reasonably constructed the dye-sensitized system of Pd/MOF and successfully extended the application of MOF to the visible range. The Pd-loaded Zr-MOF was tested for efficient photocatalytic hydrogen production and exhibited maximal photocatalytic activity (9.1 mmol/g) under visible-light irradiation (*λ* ≥ 420 nm) with EY as a photosensitizer. The remarkable enhanced properties of which was carefully studied by means of XRD, TEM, HRTEM, XPS, UV-vis diffuse reflectance, and photocatalytic hydrogen production. The results are consistent with each other. The activities of photocatalytic hydrogen production was enhanced twice order of magnitude compared with the pure MOF (0.09 mmol/g). The promotion of photocatalytic hydrogen evolution activity should be attributed to the utilization of the longer wavelength visible light and a great electronic transmission capacity of MOF. The synergistic effect between Pd and Zr-MOF is corroborated by photo-luminescence spectra and electro-chemical and photo-electro-chemical experiments, which demonstrated that the charge separation and the electron transfer are more efficient with the aid of Pd. The possible mechanism had been presented. The EY was employed for increasing absorb band gap of irradiation. UiO-66 could accept and transport electron and contribute to prolong the lifetime of charge carriers. Pd as an active species significantly enhanced the photocatalytic activity for hydrogen evolution.
